# A Frequency-Dependent Dynamic Electric–Mechanical Network for Thin-Wafer Piezoelectric Transducers Polarized in the Thickness Direction: Physical Model and Experimental Confirmation

**DOI:** 10.3390/mi14081641

**Published:** 2023-08-20

**Authors:** Lin Fa, Dongning Liu, Hong Gong, Wenhui Chen, Yandong Zhang, Yimei Wang, Rui Liang, Baoni Wang, Guiquan Shi, Xiangrong Fang, Yuxia Li, Meishan Zhao

**Affiliations:** 1 School of Information Engineering, Xi’an Fanyi University, Xi’an 710105, China; liangrui2019@126.com (R.L.); wangbn813@126.com (B.W.); 2 School of Electronic Engineering, Xi’an University of Posts and Telecommunications, Xi’an 710121, China; ldnzjs19980205@163.com (D.L.); ydzhang@mail.xidian.edu.cn (Y.Z.); wangyimei8026@163.com (Y.W.); fangxr@xupt.edu.cn (X.F.); 3 Graduate School, Xi’an University of Posts and Telecommunications, Xi’an 710121, China; gonghong@xupt.edu.cn; 4 Logging Institute, CNPC Logging, Xi’an 710077, China; chenwh20221006@126.com (W.C.); shiguiquan1@163.com (G.S.); lyx99025@163.com (Y.L.); 5 James Franck Institute and Department of Chemistry, The University of Chicago, Chicago, IL 60637, USA; m-zhao@uchicago.edu

**Keywords:** thickness direction, polarization, thin-wafer piezoelectric transducer, intrinsic noise

## Abstract

This paper is concerned with electric–acoustic/acoustic–electric conversions of thin-wafer piezoelectric transducers polarized in the thickness direction. By introducing two mechanical components with frequency-dependent values, i.e., radiation resistance and radiation mass, into the equivalent circuit of the thin-wafer piezoelectric transducer, we established a frequency-dependent dynamic mechanic-electric equivalent network with four terminals for an arbitrary given frequency, an enhancement from the conventional circuit networks. We derived the analytic expressions of its electric–acoustic and acoustic–electric conversion impulse responses using the four-terminal equivalent circuit to replace the traditional six-terminal equivalent circuit for a thin-wafer transducer with harmonic vibrational motion. For multifrequency electrical/acoustic signals acting on the transducer, we established parallel electric–acoustic/acoustic–electric conversion transmission networks. These two transmission network models have simple structures and clear physical and mathematical descriptions of thin-wafer transducers for electric–acoustic/acoustic–electric conversion when excited by a multifrequency electric/acoustic signal wavelet. The calculated results showed that the transducer’s center frequency shift relates to its mechanical load and vibration state. The method reported in this paper can be applied to conventional-sized and small-sized piezoelectric transducers with universal applicability.

## 1. Introduction

Vibration is the source of waves; a wave is a vibration state propagating through a medium. The two are closely connected and are the basis of acoustics. Thus, acoustic measurements are essential for measuring particle vibration states inside a medium.

Acoustic measurements are ubiquitous in the industry and in daily life. As an essential component in acoustic measurements, a transducer is fundamental for achieving the mutual conversion of acoustic and electric signals. A wide variety of piezoelectric transducers that are conventionally sized and small-sized are applied in many different fields, and some accompanying acoustic phenomena are investigated. In medical and biomedical fields, scientists distinguish normal and pathological issues to prevent, discover, and treat patients carrying some diseases by analyzing the interaction between human tissues and acoustic signals measured by small-sized transducers [1,2]. Liu et al. used an arrayed flexible piezoelectric micromachined ultrasonic transducer with a sandwich structure for adjuvant treatment of bone injury [3]. Rong et al. applied a piezoelectric micromachined ultrasonic transducer to wireless power transfer for implantable biomedical devices [4]. By observing and analyzing acoustic signals measured by the small-sized transducer, Hong et al. found that acoustic stress and wave resonance play a crucial role in plasma bubbles, and relevant acoustic studies offer new ways to achieve sustainable chemistry [5]. Ogawa pointed out that in measuring biological properties, acoustic effects play an essential role in designing miniaturized electronic instruments [6]. Pop demonstrated a high-data-rate intrabody communication link based on Lithium Niobate piezoelectric micromachined ultrasonic transducers [7]. For fluid acoustic sensors, some conventional-sized and small-sized piezoelectric transducers are also widely investigated for efficiently and precisely detecting the fluid’s physical properties, e.g., impurities in oil, toxic substances in sewage, viscidity variation of blood, and so on [8,9,10,11]. Zhao also researched the frequency shift induced by fluid viscosity for the frequency property of the measured acoustic signal [12]. Wang et al. performed a study on the high-frequency vibration of piezoelectric plates excited by lateral electric fields produced by surface electrodes under viscous liquid loadings for sensing [13]. For the industrial application of nondestructive detection, the acoustic signals measured by conventional-sized and small-sized transducers can provide valuable information for detecting the level of liquid carbon dioxide in containers or the oil level in a pipeline [14,15,16,17] and the quality of mechanical components [18,19,20,21,22]. The vibration responses of the objects (such as bridges, aero engines, and so on), which are measured using conventional-sized and small-sized transducers, may provide data for safety inspections, early warning against structural damage, and any types of anomalies [23,24]. For the fields of environmental protection and geological disaster prediction, using a conventional-sized transducer to perform acoustic measurements of buildings and surrounding environments can help improve hearing conditions [25,26,27]. Ambient noise measured by the conventional-sized transducer can be used to invert some valuable information, such as the inversion of underground rock dynamic velocity structure from imaging background noise for earthquake prediction [28,29,30]; moreover, because ocean noise carries some valuable marine environmental information, its acoustic inversion is essential for ocean research [31,32,33]. Conventional-sized piezoelectric transducers have also been widely used in petroleum logging and seismic exploration instruments [34,35,36]. In underwater acoustics applications, larger-sized transducers are used to detect acoustic signals propagating over a long distance for real-time tracking and the high-precision positioning of underwater targets [37,38,39].

For different purposes, either application or scientific exploration, scientists have built physical models of many different transducers with various structures, shapes, polarization modes, etc., to study their physical properties using distinct methods. Wang et al. studied interdigital transducers and provided accurate modeling of the piezoelectric effect of coupled structures [40,41,42]. Zhao et al. performed analyses on the lateral edge effect and segmented boundary conditions at a solid–liquid interface of the transducer [43]. Hara et al. proposed a structural system identification method that uses semiactive inputs generated from piezoelectric transducers [44]. Hur studied planar ultrasonic transducers based on a metasurface piezoelectric ring array for subwavelength acoustic focusing [45]. Antonino et al. developed and investigated a particular geometry of transducers, emulating the shape of bats’ cochlea, to transmit and receive ultrasounds in the air [46]. Meanwhile, Yan et al. also studied piezoelectric material‘s electric–mechanical conversion coefficient to improve the piezoelectric transducer’s electric–acoustic conversion efficiency [47]. To generate precise and guided ultrasonic wave pulses for nondestructive testing purposes, Upadhyay and Schaal conducted research on an experimental model for commonly used guided ultrasound transducers [48]. Sherrit et al. compared Mason and KLM’s equivalent circuits with six terminals for piezoelectric resonators in the thickness mode [49]. Mel’kanovich derived electric–acoustic six-terminal network equations for the transducer [50], and Sherrit and Konovalov provided a method for all transfer functions of a piezoelectric transducer with allowance for its electrical load [51]. 

The transient response of an object, e.g., a transducer, provides information on its physical property and measurement quality. Theoretical and experimental studies reported by Piquette showed that, under the excitation of a sinusoidal voltage signal, the dynamical state on the surface of a piezoelectric transducer went through a transient transition process from a static state to a steady state sinusoidal vibration [52,53]. Moon et al. found that the resonant frequency of the piezoelectric transducer changed with the load’s fluctuations and environmental conditions and proposed a method to track the resonant frequency using the transducer’s transient characteristics [54]. Wang et al. investigated the relationship between eigenfrequency and the early strength development of cement mortar by using a piezoelectric transducer [55]. Their study indicated that the eigenfrequency is related to the mechanical loading (i.e., the property of the cement mortar around the transducer) and is an integrated response from the piezoelectric transducer’s electric-acoustic (or acoustic–electric) conversions. Redwood qualitatively described the transient properties of piezoelectric transducers: “In the generation of ultrasonic waves a transient electrical signal is applied to the transducer and this produces a transient mechanical vibration, while in detection, the application of a transient mechanical signal produces an electrical vibration” [56]. On the basis of Fa and Zhao’s works [57,58,59], this paper performs a study on the physical characteristics of a thin-wafer piezoelectric transducer. The studies reported in this paper continue the earlier work with new developments and provide a mathematical description of the physical mechanism of Redwood’s research [56]. 

In studies on isotropic solids, Fa and Zhao et al. predicted that the frequency spectrum corresponding to the temporary progression of dynamical oscillations is closely related to the intrinsic noise of the transmission medium, which is related to the internal structure and physical properties of the medium, and the maximum of this frequency spectrum is determined by the solid medium’s resonant frequency and the property of external acoustic disturbance [60].

With our understanding of the physical mechanism of electric–acoustic (acoustic–electric) conversion, this paper reports a frequency-dependent dynamic electric–mechanical network for a single thin-wafer piezoelectric transducer polarized in the thickness direction by introducing radiation resistance and radiation mass [61]. The acoustic measurement goes through three processes: (i) The source transducer (referred to as the source) converts the electric-driving signal into an outward-radiated acoustic signal, and this electric–acoustic conversion process causes the vibration of particles inside the source to generate intrinsic noise; (ii) the radiated acoustic wave causes the vibration of particles inside the medium, and the interaction between the particles inside the transmission medium leads to the propagation of the acoustic wave in the medium [62], so it would also generate the intrinsic noise inside the medium [60]; (iii) the acoustic wave propagating in the medium is converted into an electric signal by the receiving transducer (referred to as the receiver), and the intrinsic noise generated by the acoustic–electric conversion will also appear. Therefore, the measured acoustic signal, i.e., the electric signal at the electric terminals of the receiver, contains intrinsic noise from the transient transition of the electric–acoustic conversion of the source: that of the vibration of particle inside the medium and that of the acoustic–electric conversion of the receiver. Specifically, to describe the physical process of acoustic measurements clearly, we established a parallel transmission network consisting of many mechanical–electric equivalent circuits for the electric–acoustic/acoustic–electric conversions of the thin-wafer transducer under the excitation of multifrequency electric/acoustic signal wavelets. The impulse responses and system functions of electric–acoustic and acoustic–electric exchanges were derived from the residue theorem for a given sinusoidal frequency. Furthermore, a new method was proposed to extract the intrinsic noise generated by the propagation medium from the integrated frequency response for transitioning from the source to the receiver. 

This study provides a theoretical basis for applying intrinsic noise to acoustic measurements. Based on the analysis of the frequency response characteristics under the excitation of multifrequency-driving signal wavelet, we discuss the physical mechanism of intrinsic noise generated by the electric–acoustic and acoustic–electric conversions of the transducer. Because any object with mass has inertia, there are transient transition processes for electric–acoustic and acoustic–electric conversions as well as the vibrations of particles inside the media. The signals obtained from acoustic measurements contain three kinds of intrinsic noises. The electric–acoustic (and acoustic–electric) conversion properties are vital to extracting noise generated inside the medium for the accurate inversion of its physical properties and internal structure. 

Studying the transient characteristics of piezoelectric transducers as a general transducer or as a fluid acoustic sensor is essential. One potential application of the research reported in this paper is that by comparing the difference between the integrated frequency responses of source–medium–receiver and source–receiver processes, the physical properties and internal structure of the medium should be inverted accurately from the measured acoustic signals.

## 2. Background Information and New Physical Model

**Figure 1:**
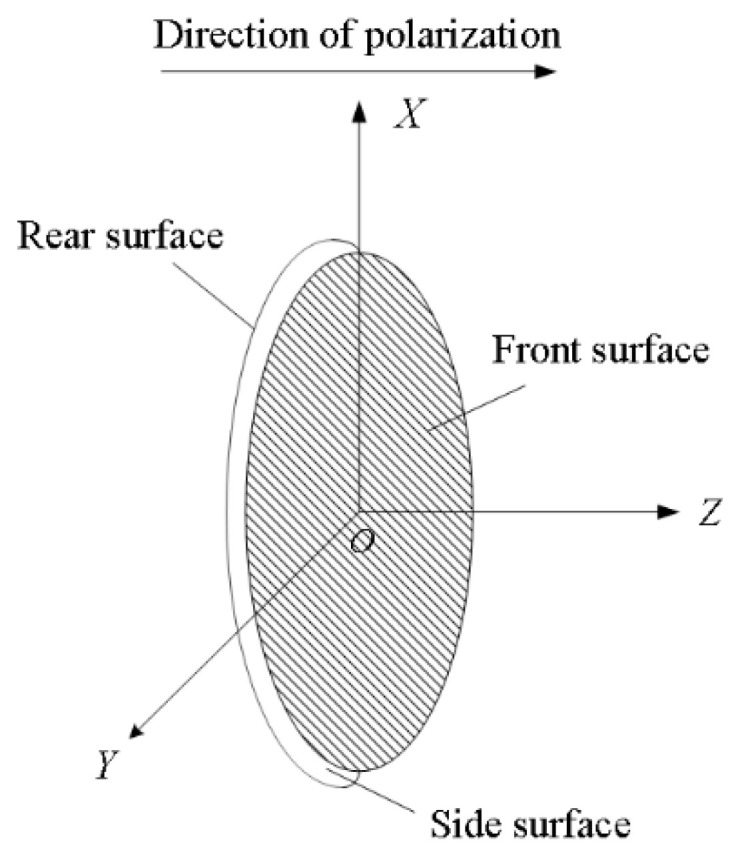
A schematic presentation of a thin-wafer transducer polarized in its thickness direction.

Let us consider a thin-wafer piezoelectric transducer in a stationary state with a given radius ($r_{0}$) and thickness ($l_{t}$), and it is polarized along the thickness direction. The two surfaces of the wafer disc of the transducer comprise two electrodes, as shown in Figure 1. Sur0, Sur1, and Sur2 refer to the side, rear, and front surfaces, respectively.

Because the thickness of the transducer is much thinner than its radius and the intrinsic vibration frequency in the thickness direction is much higher than that in the radial direction, a simplified electric field applied to the transducer in the thickness direction may be helpful. Such a transducer is in a clipped state, with no time to deform in the radial direction, and only the strain component (*S*_3_) in the *Z* direction on the *Z* surface is nonzero. 

Let us label the Cartesian coordinates (*X*, *Y*, *Z*) as (1, 2, 3). In that case, we may label the component of electric field strength in the *Z*-axis (thickness) direction ($E_{3}$) and the electric displacement vector in the *Z*-axis direction ($D_{3}$). Now, the electric field applied to the transducer is in the same direction as the polarization, and its longitudinal piezoelectric equations are

(1) $$T_{3}=C_{33}^{D}S_{3}-h_{33}D_{3}$$

(2) $$E_{3}=-h_{33}S_{3}+\beta_{33}^{S}D_{3}$$

Subscript “3” indicates the component of a physical quantity in the Z direction, such as the stress ($T_{3}=T_{ZZ}$) and strain ($S_{3}=S_{ZZ}$) and the elastic matrix element under constant electric displacement ($C_{33}^{D}=C_{ZZZZ}^{D}$). Subscript “33” means “ZZ” for the dielectric isolation ratio matrix element under constant strain ($\beta_{33}^{S}=\beta_{ZZ}^{S}$) and “ZZZ” for the piezoelectric stiffness matrix element ($h_{33}=h_{ZZZ}$), where the first “3” (in $h_{33}$) stands for “*Z*” and the second “3” stands for “*ZZ*”.

Suppose the insulation performance of a transducer is ideal (no defects nor malfunctions). Then, only the *Z*-component of the electric displacement exists ($D_{3}\neq 0$ and $\partial D_{3}/\partial Z=0$), other components will be zeroes ($D_{1}=D_{2}=0$, i.e., $D_{X}=D_{Y}=0$), and there is no free charge inside the transducer. For a given density of piezoelectric material (ρ) and the particle displacement component ($u_{Z}$), we obtain the vibrational equation of motion along the thickness (*Z*-axis) direction as

(3) $$\rho \frac{\partial^{2}u_{Z}}{\partial t^{2}}=\frac{\partial T_{3}}{\partial Z}\approx \frac{T_{3}}{l_{t}}$$

Multiplying both sides of Equation (3) by the volume of the transducer ($V=\pi r_{0}{}^{2}l_{t}$) and noting the mass of the transducer (m), we obtain

(4) $$m\frac{\partial^{2}u_{Z}}{\partial t^{2}}=T_{3}\pi r_{0}{}^{2}$$

By placing the transducer in coupling liquid and vibrating in the *Z*-direction, the surrounding medium expands and contracts alternately, and the transducer radiates acoustic waves outward, which will generate a reaction force that is exerted on the transducer.

(5) $$F_{r}=-\left(R_{r}+iX_{r}\right)\frac{du_{Z}}{dt}$$

In Equation (5), we have several related parameters, namely, radiation resistance ($R_{r}=\rho_{0}c_{0}[k^{2}r_{0}^{2}/(1+k^{2}r_{0}^{2})]S_{0}$), the radiation reactance of the transducer ($X_{r}=\rho_{0}c_{0}[kr_{0}/(1+k^{2}r_{0}{}^{2})]S_{0}$), the density of the coupling medium ($\rho_{0}$), the acoustic velocity ($c_{0}$), and wave number ($k=\omega /c_{0}$) in the coupling medium. Because the radius of the wafer is much larger than its thickness ($r_{0}>>l_{t}$) and its side and rear surfaces, i.e., Sur0 and Sur1 are in the clamped state, we only need to consider the contribution of the front surface area to radiation resistance ($R_{r}$) and radiation reactance ($X_{r}$), which means that we choose to use an effective surface ($S_{0}\approx \pi r_{0}{}^{2}$). 

Due to the viscosity of the piezoelectric material, there is frictional force resistance (*R_m_*) for the particle inside the transducer during its vibration, leading to the corresponding frictional force.

(6) $$F_{f}=-R_{m}\frac{du_{Z}}{dt}$$

The magnitude of the frictional force is related to the viscosity of the piezoelectric material, the contact area of the vibrating surface of the transducer with the coupling fluid, and the physical property of the coupling fluid.

Overall, the resultant external (electric or mechanical) disturbance acting on the transducer when vibrating in coupling liquid is

(7) $$F=F_{r}+F_{f}=-\left(R_{r}+R_{m}+iX_{r}\right)\frac{{du}_{Z}}{dt}$$

During vibrations, the transducer is affected by both external forces and self-generated internal stress. Then, we have a complete equation of motion instead of Equation (4).

(8) $$m\frac{\partial^{2}u_{Z}}{\partial t^{2}}=T_{3}\pi r_{0}{}^{2}-\left(R_{r}+R_{m}+iX_{r}\right)\frac{du_{Z}}{dt}$$

Meanwhile, from Equations (1) and (2), we have

(9) $$T_{3}=\left(C_{33}^{D}-\frac{h_{33}{}^{2}}{\beta_{33}^{S}}\right)S_{3}-\frac{h_{33}E_{3}}{\beta_{33}^{S}}$$

By the substitution of Equation (9) into Equation (8), we obtain

(10) $$m\frac{\partial^{2}u_{Z}}{\partial t^{2}}=\left(C_{33}^{D}-\frac{h_{33}{}^{2}}{\beta_{33}^{S}}\right)S_{3}\pi r_{0}{}^{2}-\frac{h_{33}E_{3}}{\beta_{33}^{S}}\pi r_{0}{}^{2}-\left(R_{r}+R_{m}+iX_{r}\right)\frac{{du}_{Z}}{dt}$$

**Figure 2:**
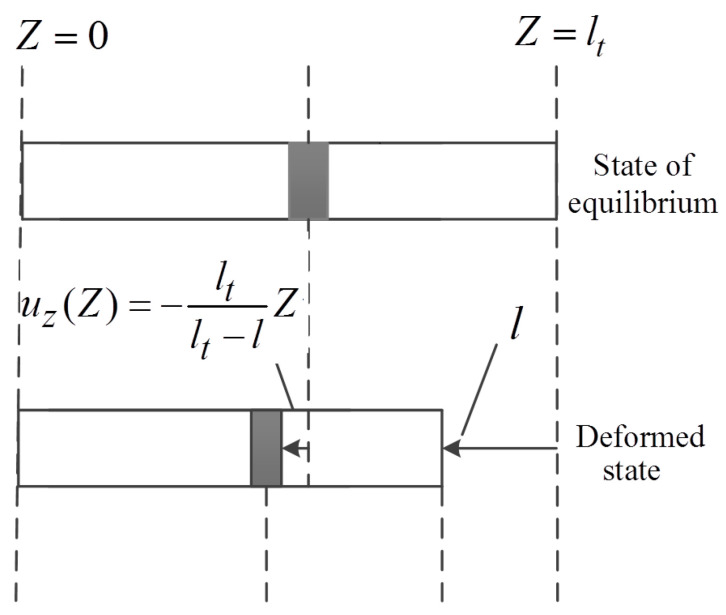
Compression/tension deformation of a uniform rod.

Combined with actual acoustic sensors, we may set the rear surface (Sur1) of the wafer transducer so that it is tightly cemented to the backing layer (i.e., this surface is considered stationary), and the front surface side (Sur2) is coated with a skinny layer of insulating material; i.e., the front surface is considered approximately free and radiates acoustic waves outward). Because the thickness of the thin-wafer transducer is much smaller than its radius, the edge effect on the establishment of the machine–electric transmission network model is negligible, and we can apply the compression/tension deformation of a uniform rod to the dynamic state of the transducer, as shown in Figure 2. 

The micro-volume element (or particle) displacement in the thickness direction (i.e., *Z*-axis direction in Figure 2) inside the thin-wafer piezoelectric transducer can be written as follows:

(11) $$u_{Z}(Z)=-\left(l_{t}-l\right)\frac{Z}{l_{t}}=-\left(1-\frac{l}{l_{t}}\right)Z$$

where *l* is the thickness of the transducer during its vibration process, and *Z* is the position of the micro-volume element inside the transducer.

The relation of the strain versus particle displacement

(12) $$S=\nabla_{s}u$$

leads to

(13) $$S_{3}=\frac{du_{Z}}{dZ}=\frac{l-l_{t}}{l_{t}}$$

We only need to consider the vibration state of the radiation surface, Sur2, and the displacement ($l-l_{t}$) created by this surface; thus, in the range inside the transducer near Sur2, Formula (13) can be rewritten as

(14) $$S_{3}=\frac{du_{Z}}{dZ}\approx \frac{Z}{l_{t}}$$

By substituting Equation (14) into Equation (10), the state equation of the transducer vibrating in the coupling liquid can be obtained.

(15) $$m\frac{d^{2}u_{Z}}{dt^{2}}+\left(R_{r}+R_{m}+iX_{r}\right)\frac{du_{Z}}{dt}-\left(C_{33}^{D}-\frac{h_{33}{}^{2}}{\beta_{33}^{S}}\right)\frac{u_{Z}\pi r_{0}{}^{2}}{l_{t}}=-\frac{\pi r_{0}{}^{2}h_{33}}{\beta_{33}^{S}}E_{3}$$

Under harmonic vibrations along the thickness direction with the amplitude, $u_{0}$, of the response, the temporal particle displacement of the transducer’s radiation surface Sur2 would be

(16) $$u_{Z}=u_{0}e^{i\left(\omega t-kZ\right)}$$

The substitution of Equation (16) into Equation (15) yields the following expression.

(17) $$u_{Z}=\frac{-\left(\pi r_{0}{}^{2}h_{33}/\beta_{33}^{S}\right)E_{3}}{-\omega^{2}\left(m+m_{r}\right)+i\omega \left(R_{r}+R_{m}\right)+\left(1/C_{m}\right)}$$

Mechanical compliance ($C_{m}=1/\left(\pi r_{0}{}^{2}(h_{33}{}^{2}/\beta_{33}^{S}-C_{33}^{D})\right)$) depends only on the intrinsic properties of the transducer. Nevertheless, radiation resistance ($R_{r}$) and radiation quality ($m_{r}=X_{r}/\omega$) are related to the geometric size of the transducer, the physical property of the coupling medium, and the frequency of the sinusoidal signal (electric or acoustic) that excites the transducer. 

Based on Gauss’s theorem, the total charge on each electrode is as follows [63].

(18) $$Q=\iint D_{3}dA=D_{3}\pi r_{0}{}^{2}$$

A combination of Equations (2) and (14) yields

(19) $$D_{3}=(E_{3}/\beta_{33}^{S})+(h_{33}u_{Z}/\beta_{33}^{S}l_{t})$$

The instantaneous current comes from the first-order derivation of the accumulated charge on each electrode surface concerning time. The substitution of Equations (17) and (19) into Equation (18) yields the instantaneous current.

(20) $$I=\frac{dQ}{dt}=i\omega Q=i\omega C_{0}V+\phi^{2}\frac{V}{\left(R_{m}+R_{r}\right)+i\omega \left(m+m_{r}\right)+1/i\omega C_{m}}$$

Equation (20) is also known as the mechanical–electric state equation of the transducer, where several parameters are the static capacitance of the transducer ($C_{0}=\pi r_{0}{}^{2}/\beta_{33}^{S}l_{t}$), the voltage applied between the two electrodes ($V=E_{3}l_{t}$), and the mechanical–electric conversion coefficient ($\phi =\pi r_{0}{}^{2}h_{33}/\beta_{33}^{S}l_{t}$).

**Figure 3:**
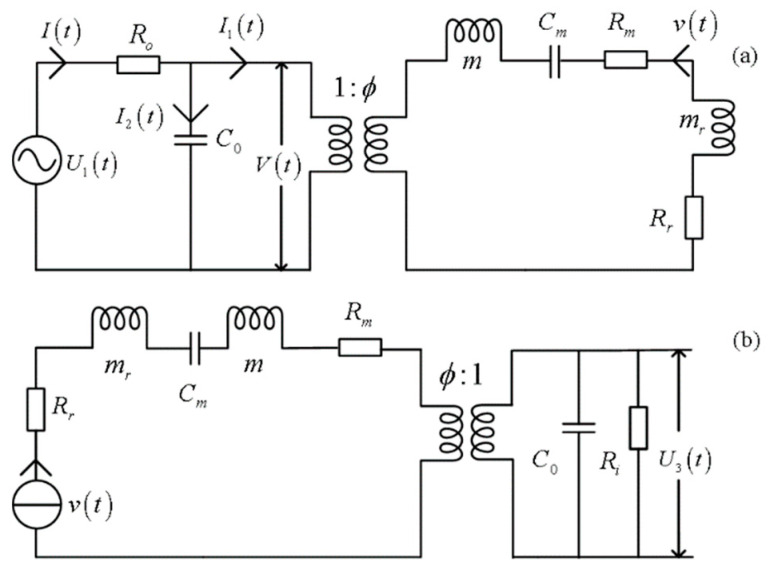
The time-domain mechanical–electric equivalent circuits of a transducer: (**a**) the transducer as a source; (**b**) the transducer as a receiver.

We can establish the mechanical–electric equivalent circuits of the electric–acoustic and acoustic–electric conversions of the transducer based on Equation (20), as shown in Figure 3. The defined physical quantities in Figure 3 are the sinusoidal driving-voltage signal ($U_{1}(t)$), the output resistance of the driving-voltage source ($R_{o}$), the voltage signal at the electric terminal of the receiver ($U_{3}(t)$), the input resistance of the measurement circuit ($R_{i}$), and the particle displacement velocity on the surface of the transducer (v(t)). 

We must pay attention to substantial differences in the characteristics and the analysis methods between conventional circuits and the mechanical–electric equivalent circuit of the transducer. The values of all components, e.g., resistance and inductance, etc., are independent of frequency in conventional circuits. In contrast, some mechanical elements in the mechanical–electric equivalent electric circuits of the transducer are functions of frequency, e.g., radiation resistance and radiation quality, as shown in Figure 3.

**Figure 4:**
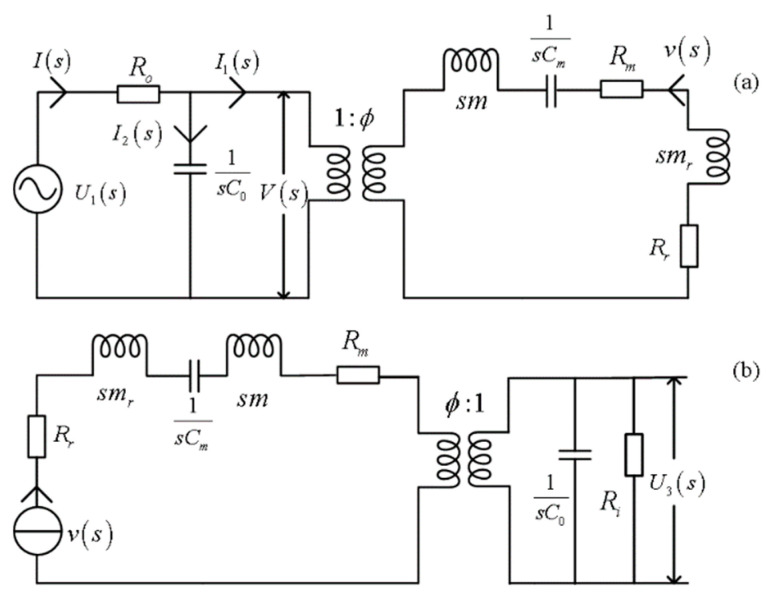
The s-domain mechanical–electric equivalent circuits of the transducer: (**a**) source; (**b**) receiver.

The mechanical–electric equivalent circuits in the time domain, as shown in Figure 3, were transformed into the *s* domain, as shown in Figure 4. From there, we analyze the inherent characteristics of the transducer and solve its electric–acoustic (and acoustic–electric) impulse responses and the corresponding electric–acoustic (and acoustic–electric) system functions. 

From the equivalent mechanical–electric circuit of the source, as shown in Figure 4a, and based on Kirchhoff’s law, we have

(21) $$U_{1}(s)=V(s)+I(s)R_{o}$$

(22) $$I(s)=I_{1}(s)+I_{2}(s)=\phi v(s)+sC_{0}V(s)$$

Since the initial state of the transducer is at equilibrium, the initial energy storage of the relevant electric and mechanical (or dynamical) components (such as capacitance, mass, radiation mass, compliance, etc.) in the mechanical–electric circuit is zero. Any response would have to be from external excitation, and the processes of electric–acoustic (and acoustic–electric) conversions would define the zero-state response. Therefore, in the *s* domain, the system function of electric–acoustic conversions is a simple ratio of the surface vibration velocity (*v*(*s*)) of the transducer to the driving voltage (*U*_1_(*s*)). And from Equations (21) and (22), we have

(23) $$H_{1}(s)=\frac{v(s)}{U_{1}(s)}=\frac{1}{R_{o}\phi +\left(R_{1}\left(1+sC_{0}R_{o}\right)/\phi \right)}$$

where $R_{1}=\phi V(s)/v(s)=s\left(m+m_{r}\right)+\left(R_{m}+R_{r}\right)+1/sC_{m}$.

Let us define a few parameters to simplify the discussion and mathematical derivation: $a=1/C_{0}R_{o}+\left(R_{m}+R_{r}\right)/\left(m+m_{r}\right)$, $b=\left(\left(R_{m}+R_{r}\right)/C_{0}R_{o}+1/C_{m}+\phi^{2}/C_{0}\right)/\left(m+m_{r}\right)$, $c={\left(\left(m+m_{r}\right)C_{0}C_{m}R_{o}\right)}^{-1}$, and $d=\left(\phi /\left(m+m_{r}\right)C_{0}R_{o}\right)$. Then, we can write Equation (23) as

(24) $$H_{1}(s)=\frac{ds}{s^{3}+as^{2}+bs+c}$$

Its discriminant ($D=p^{3}+q^{2}={\left(b/3+a^{2}/9\right)}^{3}+{\left(a^{3}/27-ab/6+c/2\right)}^{2}$) controls the mode of dynamical motion. 

Based on the residue theorem, we would have the following cases of the impulse response for the electric–acoustic conversion:

(i) The discriminant has a negative value (D<0), and system function ($H_{1}(s)$) has three non-equal real poles.

(25) $$s_{1}=-\alpha$$

(26) $$s_{2}=-\beta +\sqrt{3}B$$

(27) $$s_{3}=-\beta -\sqrt{3}B$$

The relevant parameters are α=a/3−2A, β=a/3+A, A=(x+y)/2, B=(x−y)/2, $x={\left(-q+\sqrt{D}\right)}^{1/3}$, and $y={\left(-q-\sqrt{D}\right)}^{1/3}$. In this case, employing a unit step function (ε(t)), the impulse response from the electric–acoustic conversion of the source is

(28) $$h_{1}(t)=A_{1}e^{-\alpha t}\varepsilon (t)+e^{-\beta t}\left[B_{1}ch\left(\sqrt{3}\beta t\right)+C_{1}sh\left(\sqrt{3}\beta t\right)\right]\varepsilon (t)$$

This presents a state of over damping, where $A_{1}=-d\alpha /\left[{\left(\alpha -\beta \right)}^{2}-3B^{2}\right]$, $B_{1}=d\alpha /\left[{\left(\alpha -\beta \right)}^{2}-3B^{2}\right]$, and $C_{1}=\left(d/\sqrt{3}B\right)\left(\beta (\beta -\alpha )-3B^{2}\right)/\left({\left(\alpha -\beta \right)}^{2}-3B^{2}\right)$.

(ii) The discriminant is zero (D=0), and the system function ($H_{1}\left(s\right)$) has three real poles, two of which are identical.

(29) $$s_{1}=-\alpha$$

(30) $$s_{2}=-\beta$$

(31) $$s_{3}=-\beta$$

The electric–acoustic conversion impulse response is

(32) $$h_{1}(t)=A_{2}e^{-\alpha t}\varepsilon (t)+\left(B_{2}t+C_{2}\right)e^{-\beta t}\varepsilon (t)$$

where $A_{2}=-d\alpha /{\left(\beta -\alpha \right)}^{2}$, $B_{2}=d\beta /\left(\beta -\alpha \right)$, and $C_{2}=d\alpha /{\left(\beta -\alpha \right)}^{2}$. Equation (32) shows that the transducer is in a critical damping state. 

(iii) The discriminant has a positive value (D>0), and the system function ($H_{1}\left(s\right)$) has a real-valued pole and a pair of complex conjugate poles.

(33) $$s_{1}=-\alpha$$

(34) $$s_{2}=-\beta +i\sqrt{3}B_{3}$$

(35) $$s_{3}=-\beta -i\sqrt{3}B_{3}$$

The impulse response of the electric–acoustic conversion is in an oscillatory mode with an oscillation frequency, $f_{1s}=\omega_{1s}/2\pi$, which can be written as

(36) $$h_{1}(t)=A_{3}e^{-\alpha t}\varepsilon (t)+B_{3}e^{-\beta t}\cos\left(\omega_{1s}t+\theta \right)\varepsilon (t)$$

(37) $$\omega_{1s}=\sqrt{3}B$$

where $A_{3}=-d\alpha /\left(\sigma^{2}+3B^{2}\right)$, $B_{3}=2{\left(M^{2}+N^{2}\right)}^{1/2}$, σ=β−α, $\theta =\arctan\left(N/M\right)$, $M=d\alpha /\left(\sigma^{2}+3B^{2}\right)$, and $N=-d(\beta \sigma +3B^{2})/\sqrt{3}B(\sigma^{2}+3B^{2})$. In this case, the source is in an under-damped state, and the radiated acoustic waves proceed outwards under the excitation of the driving-voltage signal, in line with the actual physical condition. The source’s oscillation frequency is related to the radiation resistance and mass, so it is an important physical quantity for describing the characteristics of the transient process generated by the transducer under the excitation of any sinusoidal voltage signal with a given frequency.

Since Equation (36) is integrable with an existing relation (s=iω), the electric–acoustic conversion system function of a source, shown in Equation (24), yields

(38) $$H_{1}(i\omega )={\left.H_{1}(s)\right|}_{s=i\omega }=\frac{i\omega d}{-i\omega^{3}-\omega^{2}a+i\omega b+c}$$

Similarly, acoustic–electric conversion is the reverse of electric–acoustic transformation for a transducer. The system function of acoustic–electric conversion for the receiver is the ratio of voltages between its two electrodes to the particle displacement velocity on its surface in the frequency domain. 

Since the transducer works in the oscillation mode, we only need to discuss the under-damped state of the receiver. Similarly to the source’s electric–acoustic conversion, we can obtain the acoustic–electric impulse response, the system function of the acoustic–electric conversion, and the oscillation angular frequency of the receiver.

(39) $$h_{3}(t)={A^{\prime }}_{3}e^{-\alpha^{\prime }t}\varepsilon (t)+{B^{\prime }}_{3}e^{-\beta^{\prime }t}\cos\left(\omega_{3s}t+\theta^{\prime }\right)\varepsilon (t)$$

(40) $$H_{3}(i\omega )=\frac{i\omega d^{\prime }}{-i\omega^{3}-\omega^{2}a^{\prime }+i\omega b^{\prime }+c^{\prime }}$$

(41) $$\omega_{3s}=\sqrt{3}B^{\prime }$$

Here, we have $a^{\prime }=/C_{0}R_{i}+\left(R_{m}+R_{r}\right)/\left(m+m_{r}\right)$, $b^{\prime }=\left(\left(R_{m}+R_{r}\right)/C_{0}R_{i}+1/C_{m}+\phi^{2}/C_{0}\right)/\left(m+m_{r}\right)$, $c^{\prime }=\left(1/C_{m}+\phi^{2}/C_{0}\right)/\left(m+m_{r}\right)R_{i}C_{0}$, $d^{\prime }=\rho_{0}c_{0}\phi /(m+m_{r})C_{0}$, $p^{\prime }=b^{\prime }/3+{a^{\prime }}^{2}/9$, $q^{\prime }={a^{\prime }}^{3}/27-a^{\prime }b^{\prime }/6+c^{\prime }/2$, $D^{\prime }={p^{\prime }}^{3}+{q^{\prime }}^{2}$, $x^{\prime }={\left(-q^{\prime }+\sqrt{D^{\prime }}\right)}^{1/3}$, $y^{\prime }={\left(-q^{\prime }-\sqrt{D^{\prime }}\right)}^{1/3}$, $A^{\prime }=\left(x^{\prime }+y^{\prime }\right)/2$, $B^{\prime }=\left(x^{\prime }-y^{\prime }\right)/2$, $\alpha^{\prime }=a^{\prime }/3-2A^{\prime }$, $\beta^{\prime }=a^{\prime }/3+A^{\prime }$, $\sigma^{\prime }=\beta^{\prime }-\alpha^{\prime }$, $A_{3}{}^{\prime }=-d^{\prime }\alpha^{\prime }/\left({\sigma^{\prime }}^{2}+3{B^{\prime }}^{2}\right)$, $B_{3}{}^{\prime }=2{\left({M^{\prime }}^{2}+{N^{\prime }}^{2}\right)}^{1/2}$, $M^{\prime }=d^{\prime }\alpha^{\prime }/\left({\sigma^{\prime }}^{2}+3{B^{\prime }}^{2}\right)$, $N^{\prime }=-d^{\prime }(\beta^{\prime }\sigma^{\prime }+3{B^{\prime }}^{2})/\sqrt{3}B^{\prime }({\sigma^{\prime }}^{2}+3{B^{\prime }}^{2})$, and $\theta^{\prime }=\arctan\left(N^{\prime }/M^{\prime }\right)$.

## 3. Calculation and Analysis

In the following discussion and calculations, we selected water as the coupling liquid around the transducer with the known acoustic velocity ($c_{0}=1500\;m/s$) and density ($\rho_{0}=1000\;kg/m^{3}$). Table 1 displays the physical parameters of the piezoelectric material PZT-5H used for the transducer [64].

### 3.1. Structure Relationships of Oscillation and Center Frequencies versus Geometric Size

We selected the output resistance ($R_{o}$) of the driving-voltage signal source as 50 Ω, the input resistance ($R_{i}$) of the measuring circuit as 200 Ω, the free-load transducer is in a vacuum ($R_{r}=m_{r}=R_{m}=0$), and the loaded transducer is in water ($R_{m}=0.2\times \pi r_{b}^{2}\rho_{m}$). We also defined the transducer’s center frequency as the frequency corresponding to its system function’s maximum modulus. For the case of free-load, the center frequency (a source or a receiver) is irrelevant to the sinusoidal frequency of the excitation (electric or acoustic) exerted on the transducer, and it is only determined by the transducer’s intrinsic physical and geometrical properties due to $R_{r}=0$ and $m_{r}=0$. The loading center frequency relies on the transducer’s physical and geometrical parameters, the coupling medium’s physical nature, and the sinusoidal frequency of the excitation signal (either electric or acoustic).

**Figure 5:**
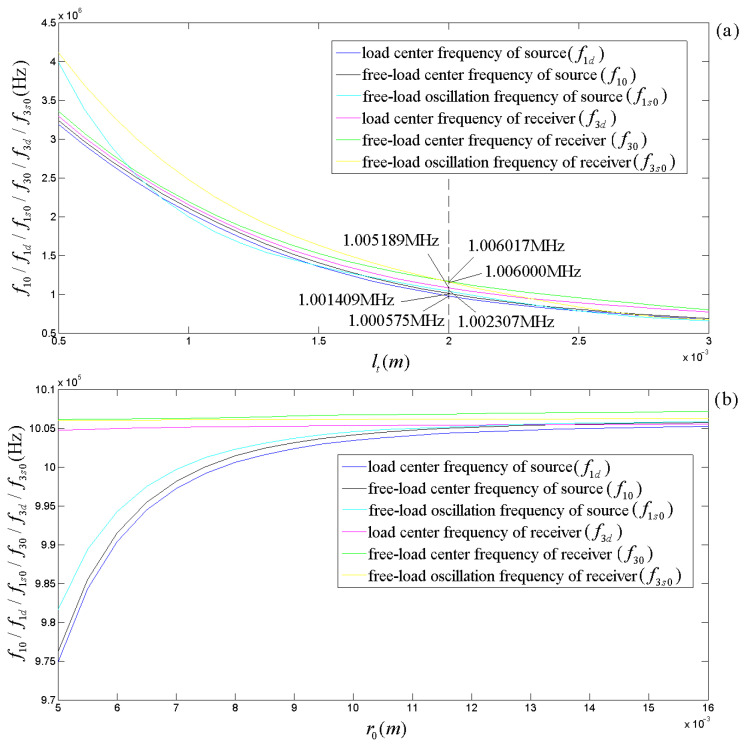
Relationships of the free-load center, load center, and free-load oscillation frequencies versus the geometric dimension of the transducer: (**a**) frequencies and thickness; (**b**) frequencies and radius.

Taking the radius ($r_{0}$) of the transducer to be 8 mm as an example, Figure 5a displays the free-load center, load center, and free-load oscillation frequencies of the transducer versus its thickness. Figure 5a shows that regardless of whether the transducer is a source or a receiver, (i) the above three kinds of frequencies decrease significantly with increasing thickness. (ii) The free-load center, load center, and oscillation frequencies of the receiver are slightly higher than those of the source. (iii) The free-load center frequency is slightly higher than the load center frequency. (iv) The oscillation frequency can be greater than the free-load center frequency within a radius range, and it can also be lower than the latter in another radius range; this is because the effect of the radius on the direct current of the impulse response (electric–acoustic or acoustic–electric) is smaller than that of the oscillation frequency for the first case, and the effect of the radius on the direct current of the impulse response is more significant than the oscillation frequency for the second case. At $l_{t}=$ 2 mm and $r_{0}=$ 8 mm, $f_{10}=1.001409$ MHz, $f_{1d}=$ 1.000575 MHz, $f_{1s0}=$ 1.002307 MHz, $f_{30}=$ 1.006017 MHz, $f_{3d}=$ 1.005189 MHz, and $f_{3s0}=$ 1.006000 MHz.

Similarly, in selecting the thickness of the transducer to be 2 mm, Figure 5b displays the free-load center, load center, and free-load oscillation frequencies of the transducer versus its radius. Figure 5b shows: (i) as a source, the free-load center, load center, and free-load oscillation frequencies of the transducer increase slightly with the radius; (ii) as a receiver, the above three kinds of frequencies of the transducer increase very slowly with increasing its radius. 

**Figure 6:**
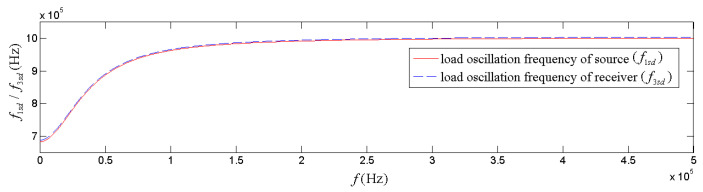
Relationships of the load oscillation frequency versus the frequency of sinusoidal (electric or acoustic) signal exerted on the transducer.

Figure 6 is the calculated relationships of load oscillation frequencies ($f_{1sd}$ and $f_{3sd}$) of both source and receiver versus the sinusoidal frequency (*f*) of the excitation signal exerted on the transducer (electric or acoustic). It displays $f_{1sd}$ and $f_{3sd}$ has a monotonic increasing tendency with both the frequency of the sinusoidal driving-voltage signal and that of the acoustic signal arriving at the receiver. It also shows that the sinusoidal signal frequency has a more significant effect on the load oscillation frequency in the lower frequency range and has a minimal impact on the load oscillation frequency in the higher frequency range. The loading oscillation frequency of the transducer (as a source or as a receiver) approaches its free-load center frequency as the frequency of the applied sinusoidal signal increases. The load oscillation frequency of the source signal is slightly smaller than that of the receiver.

### 3.2. Electric–Acoustic and Acoustic–Electric Impulse Responses and Corresponding System Functions

**Figure 7:**
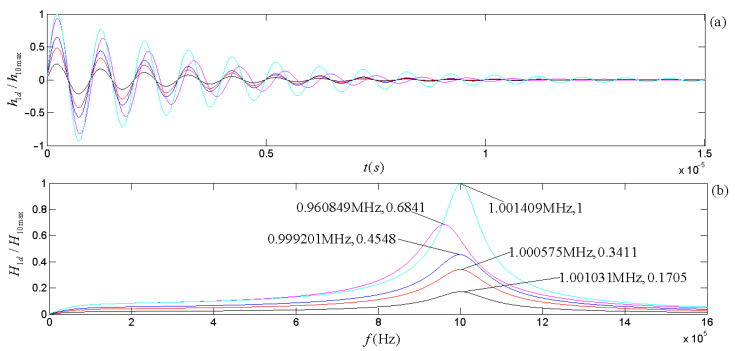
The electric–acoustic impulse responses of the source and the corresponding amplitude spectra: (**a**) electric–acoustic impulse responses; (**b**) amplitude spectra. We normalized the electric–acoustic impulse responses and amplitude spectra using the maximum values of the impulse response and amplitude spectrum for the case of free-loading, respectively. The cyan curve is the electric–acoustic impulse response and the corresponding amplitude spectrum for free-loading. The other colors are those with mechanical loads: the lines in magenta, blue, red, and black are the transducer’s electric–acoustic impulse responses and the corresponding amplitude spectra at selected frequencies of 0.1 $f_{1d}$, 0.5 $f_{1d}$, 1.0 $f_{1d}$, and 10 $f_{1d}$, respectively. Here, $f_{1d}$ = 1.000575 MHz.

**Figure 8:**
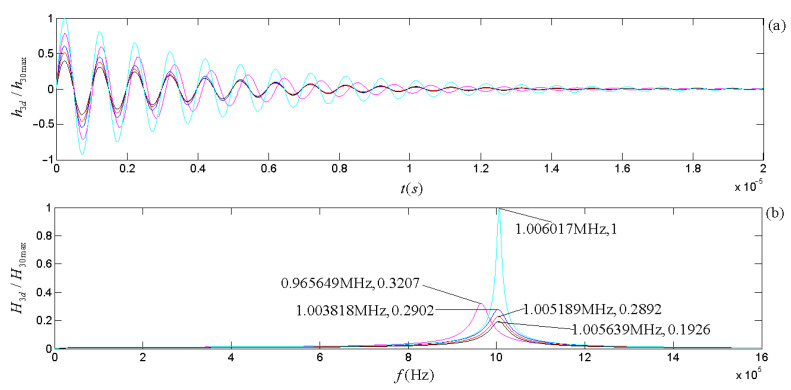
The acoustic–electric impulse responses of the receiver and the corresponding amplitude spectra: (**a**) acoustic–electric impulse responses; (**b**) amplitude spectra. We have normalized acoustic–electric impulse responses and amplitude spectra using the maximum value of the impulse response and amplitude spectrum for the case of free load, respectively. The cyan curve is the acoustic–electric impulse response and the corresponding amplitude spectrum for the free load. The other colors are those with mechanical load: the lines in magenta, blue, red, and black are the transducer acoustic–electric impulse responses, and the amplitude spectra of the corresponding system functions at selected frequencies of 0.1 $f_{3d}$, 0.5 $f_{3d}$, 1.0 $f_{3d}$, and 10 $f_{3d}$. Here, $f_{3d}$ = 1.005189 MHz.

The piezoelectric physical nature and geometric parameters of the transducers guarantee the system is always in an oscillatory mode in practical applications. Figure 7 and Figure 8 show the calculated electric–acoustic (and acoustic–electric) impulse responses and the corresponding amplitude spectra for the piezoelectric transducers (both free-load and load), which are the same as discussed in the Section 5.

Figure 3 shows that the radiation resistance and mass in the equivalent circuits are frequency-dependent. Figure 7 and Figure 8 show the calculated electric–acoustic (and acoustic–electric) impulse responses and corresponding amplitude spectra at four selected frequencies with radiation resistance and radiation mass. Interestingly, the electric–acoustic (and acoustic–electric) conversion impulse responses, the corresponding amplitude spectra, and the center frequencies are unvaried with respect to frequency for a free-load transducer but vary with the frequency for loaded transducers. It is plausible that the radiation resistance and mass of the transducer are indeed functions of frequency. The frequency spectrum corresponding to the transient process mainly reflects the inherent physical characteristics of the transducer, even though the sinusoidal signal (electric or acoustic) exerted on the transducer has some influence on it. The frequency corresponding to the amplitude spectrum’s maximum stands for the central frequency of the transducer. 

From Figure 7 and Figure 8, we observed the following: (i) The center frequencies corresponding to the electric–acoustic and acoustic–electric conversions of the transducer increase with the sinusoidal frequency and tend toward the corresponding free-load center-frequency, but the magnitudes of the corresponding amplitude spectra decrease with increasing frequency, namely the transducer’s electric–acoustic and acoustic–electric conversion efficiencies decrease with increasing frequency. (ii) For the same transducer, although its acoustic–electric conversion is the inverse process of the electric–acoustic conversion and the difference between the center frequency corresponding to electric–acoustic conversion and that corresponding to acoustic–electric conversion is also insignificant, the characteristics of its two kinds of conversions are different. For example, the bandwidth of the amplitude spectrum of the electric–acoustic conversion is greater than that of its acoustic–electric conversion; that is to say that compared with the filtering effect of the transducer on the frequency component of the driving-voltage signal far from its center frequency, the acoustic–electric conversion has a more substantial filtering effect on the frequency component of the received acoustic signal far from its center frequency.

### 3.3. Acoustic Signals Radiated by Transducers under the Excitation of Sinusoidal Driving-Voltage Signals with Different Frequencies

**Figure 9:**
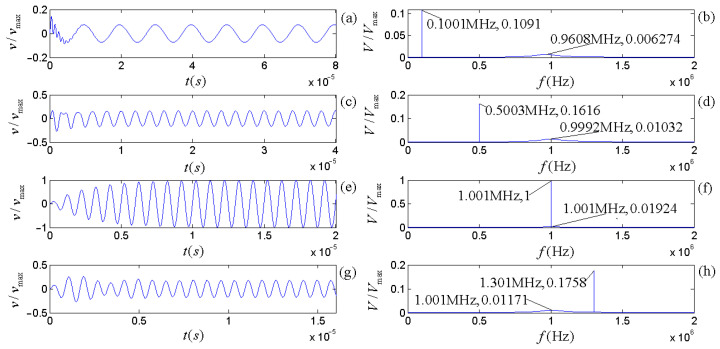
Surface vibration velocity and corresponding amplitude spectrum of the source excited by sinusoidal driving-voltage signals with different frequencies: (**a**,**c**,**e**,**g**) are the waveforms in the time domain; (**b**,**d**,**f**,**h**) are the corresponding amplitude spectra.

Four sinusoidal driving-voltage signals with an amplitude of 1 V are selected, and their four frequencies are selected as 0.1 $f_{1d}$, 0.5 $f_{1d}$, 1 $f_{1d}$, and 1.3 $f_{1d}$ to excite the source ($f_{1d}$ = 1.000575 MHz). Figure 9 shows the calculated vibration velocity of the source’s surface.

The calculated results show that when excited by any sinusoidal driving-voltage signal with a given frequency, the transducer will go through a transient process from a static state to a steady sinusoidal vibration state due to the inertia of the particles with mass inside the transducer. When the frequency of the sinusoidal driving-voltage signal is equal to the load center frequency of the source, the source resonates with the sinusoidal driving-voltage signal, and the amplitude of its surface vibration is the largest. This also means that the amplitude of the acoustic wave signal radiated outward by the source is the largest (see Figure 9e). In this case, the frequency spectrum of the sinusoidal driving-voltage signal is stacked with the frequency spectrum of the electric–acoustic conversion precisely at the load center frequency of the source (see Figure 9f). When the frequency of the sinusoidal driving-voltage signal is not equal to (greater than or less than) the load center frequency of the source, the amplitude of the radiated acoustic signal decreases.

### 3.4. The Electric Signal Is Converted from an Acoustic Signal by the Receiver Transducer

As mentioned in the earlier section, the acoustic–electric conversion of the transducer is an inverse process of the electric–acoustic transformation but differs in some characteristics. Below, we will analyze and discuss two cases.

#### 3.4.1. Case 1

**Figure 10:**
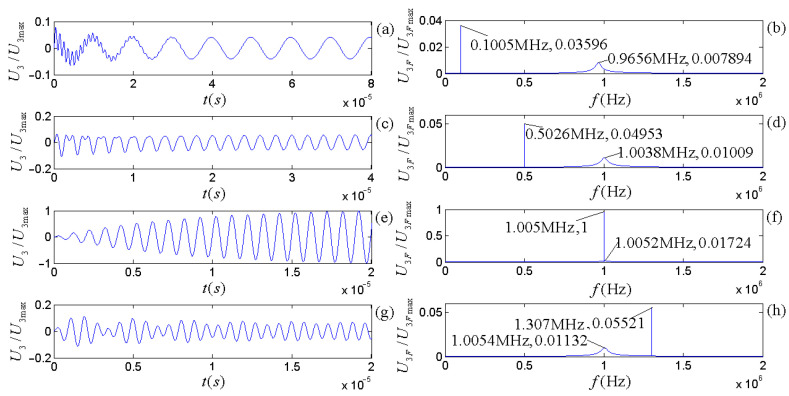
Electric signals converted from sinusoidal acoustic signals with different frequencies: (**a**,**c**,**e**,**g**) are the waveforms in the time domain; (**b**,**d**,**f**,**h**) are the corresponding amplitude spectra.

To analyze only the acoustic–electric conversion characteristics of the transducer, sinusoidal acoustic signals with an amplitude of 1 V and frequencies of 0.1 $f_{3d}$, 0.5 $f_{3d}$, $f_{3d}$, and 1.3 $f_{3d}$ are directly input into the mechanical terminals of the receiver. Here, the central frequency is $f_{3d}$ = 1.005189 MHz. Figure 10 shows the voltage signal outputted by the electric terminals of the receiver after acoustic–electric conversion.

The calculated results showed a transient process from a zero state to a steady sinusoidal voltage signal at the receiver’s electric terminals. The temporary duration of the acoustic–electric conversion is longer than the electric–acoustic conversion because the amplitude spectrum’s bandwidth corresponding to the transducer’s acoustic–electric conversion is narrower than the corresponding electric–acoustic conversion.

#### 3.4.2. Case 2

For the integrated response characteristics of the source–receiver, we set both the source and the receiver in an ideal liquid (viscosity and acoustic attenuation coefficients are zeroes) with an acoustic velocity of 1500 m per second, and the distance from the source transducer to the receiver is 0.15 m.

**Figure 11:**
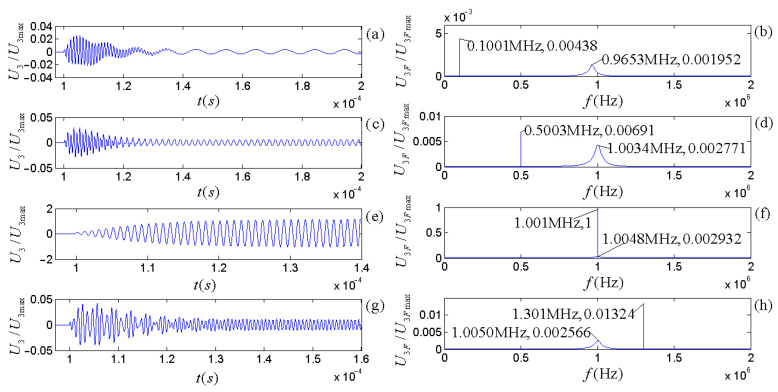
Waveform and amplitude spectrum of electric signals at the receiver’s electric terminals for the integrated response of the source–receiver: (**a**,**c**,**e**,**g**) are the waveforms in the time domain; (**b**,**d**,**f**,**h**) are the corresponding amplitude spectrum in the frequency domain.

When excited by sinusoidal driving-voltage signals with an amplitude of 1 V and various frequencies (0.1 $f_{1d}$, 0.5 $f_{1d}$, 1.0 $f_{1d}$, and 1.3 $f_{1d}$), the source radiates acoustic waves that propagate to the receiver without distortion and attenuation through the ideal liquid. Figure 11 shows the voltage signals at the receiver’s electric terminals.

Figure 11 shows the following: (i) Despite the fact that the bandwidth of the receiver’s frequency response (amplitude spectrum) is narrower than the source’s frequency response, the center frequency of the source is slightly lower than that of the receiver. (ii) When the frequency of the sinusoidal driving-voltage signal is close to the center frequency of the integrated frequency response of the source–receiver, the transient process of the electric signal at the receiver’s electrical terminals experiences the lowest number of oscillation periods, and the amplitude spectrum of the sinusoidal driving-voltage signal is superimposed on the amplitude spectrum corresponding to the integrated frequency response of the source–receiver. (iii) If the frequency of the sinusoidal driving-voltage signal is far from the center frequency of the integrated frequency response of the source–receiver, the number of periods of the electric signal at the receiver’s electric terminals from the zero state to the stable sinusoidal vibration state increases, and the amplitude of the time domain waveform and the magnitude of amplitude spectrum decreases.

### 3.5. Multifrequency Driving-Voltage Signal Exciting Transducer

To understand the comprehensive response characteristics of the source–receiver under the excitation of the wavelet of the driving-voltage signal with many different frequency components, we ignore the influence of the medium on the radiated sound wave. Assuming that the medium is ideal and elastic, the outer surfaces of the source and receiver transducer are tightly bonded to the medium, and the propagation process of the acoustic wave does not exhibit attenuation and dispersion.

**Figure 12:**
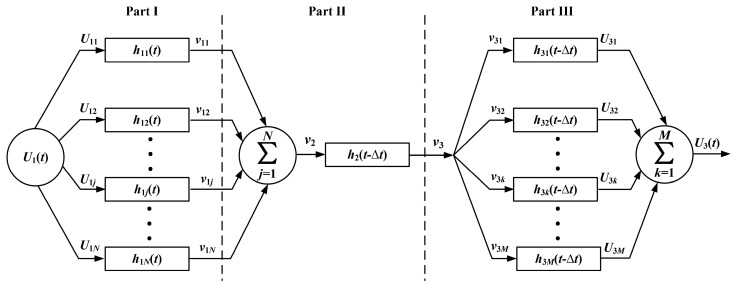
A schematic representation of a parallel transmission network of an acoustic measurement process.

In most acoustic measurements, both the driving-voltage signal exciting the source and the acoustic signal arriving at the receiver are wavelets containing many frequency components with different amplitudes and initial phases. Based on the Fourier transform, an electric (or acoustic) signal used to excite a transducer can be a linear composition of sinusoidal components with different amplitudes, frequencies, and initial phases. Because radiation impedance and mass are frequency functions, each frequency component corresponds to a different electric–acoustic (or acoustic–electric) equivalent circuit within a circuit network. The electric–acoustic (and acoustic–electric) conversion can be achieved through a parallel transmission network consisting of many electric–acoustic (and acoustic–electric) conversion circuits, as shown in Figure 12 (Parts I and III). Each electric circuit within the network would contribute its unique electric–acoustic (or acoustic–electric) impulse response due to its self-radiation resistance and mass.

In this network, the related physical parameters include the *j*^th^ frequency component ($U_{1j}$) in the driving-voltage signal ($U_{1}$) (*j* = 1, 2, …… *N*); the electric–acoustic impulse response ($h_{1j}(t)$) of the source corresponding to the *j*^th^ sinusoidal frequency component in the driving-voltage signal ($U_{1}$); the particle displacement velocity ($v_{1j}$) when excited by the sinusoidal component ($U_{1j}$) in the driving-voltage signal; the particle displacement velocity ($v_{2}$) when excited by the driving-voltage signal ($U_{1}$); the impulse response ($h_{2}(t-\Delta t)$) of the medium, the propagation time (Δt) of the acoustic wave from the source to the receiver; the acoustic signal ($v_{3}$) reaching the receiving transducer; the *k*^th^ frequency component ($v_{3k}$) (*k* = 1, 2, …… *M*) of a sound wave signal; the acoustic–electricity impulse response ($h_{3k}(t-\Delta t)$) of the receiving transducer corresponding to the *k*^th^ sinusoidal frequency component in the acoustic signal ($v_{3}$); output electric signal ($U_{3k}$) of the receiving transducer after receiving the *k*^th^ frequency component of the sound wave; and measurement of electric signals ($U_{3}$) during acoustic measurement process.

When a frequency component of a driving-voltage signal acts on a source transducer, the signal radiated from the source transducer contains more than just the frequency component corresponding to the frequency component of the driving-voltage signal. It also includes some frequency components related to the transient process of electric–acoustic conversion. On the receiver, likewise, when each frequency component of the acoustic signal transmitted from the medium acts on the receiver, due to the existence of the transient process of acoustic–electric conversion, the electric signal obtained at the receiver’s electric terminals contains not only the frequency component corresponding to the frequency component in the acoustic signal but also some frequency components corresponding to the transient acoustic–electric process. 

For each frequency component ($\omega_{j}$) of the driving-voltage signal ($U_{1}(t)$) with an amplitude spectrum ($U_{1F}(\omega )$) and phase spectrum (ϕ(ω)), we may write it as

(42) $$U_{1j}(t)=\left|U_{1F}(\omega_{j})\right|\cos\left[\omega_{j}t+\phi (\omega_{j})\right]$$

Then, we have the normalized driving-voltage signal.

(43) $$U_{1n}(t)=\sum_{j=1}^{N}U_{1j}(t)/\max[|\sum_{j=1}^{N}U_{1j}(t)|]$$

We can mathematically express the output of the *j*^th^ equivalent circuit in the first part of Figure 12.

(44) $${\left.v_{1j}(t)\right|}_{\omega_{j}}={\left.\left[U_{1j}(t)*h_{1j}(t)\right]\right|}_{\omega_{j}}$$

And we also have the normalized expression of the source’s surface vibration velocity.

(45) $$v_{2n}(t)=\sum_{j=1}^{N}{\left.v_{1j}(t)\right|}_{\omega_{j}}/\max\left(|\sum_{j=1}^{N}{\left.v_{1j}(t)\right|}_{\omega_{j}}|\right)$$

The acoustic signal reaching the receiving transducer is

(46) $$v_{3}(t)=v_{2}(t)*h_{2}(t-\Delta t)$$

The output electric signal of the receiving transducer after receiving the *k^th^* frequency component of the sound wave is

(47) $$U_{3k}(t)={\left.\left[v_{3k}(t)*h_{3k}(t-\Delta t)\right]\right|}_{\omega_{k}}$$

The cumulative outputs of all circuits in Part III of Figure 12, i.e., the electric signal wavelet at the receiver’s electric terminals, can be expressed by

(48) $$U_{3}(t)=\sum_{k}^{M}U_{3k}(t)$$

Finally, the normalized overall output signal is

(49) $$U_{3n}(t)=U_{3}(t)/\max[U_{3}(t)]$$

Now, let us consider the following gate-selection sinusoidal signal as the driving-voltage signal of the excitation source with the specific amplitude ($U_{0}$), angular frequency ($\omega_{g}$), and time window ($t_{0}$). We define the Heaviside function as (H(⋅)), and we use the signal to perform some calculations.

(50) $$U_{1}(t)=\left[H(t)-H(t-t_{0})\right]U_{0}\sin(\omega_{g}t)$$

The driving-voltage signal in the frequency domain is

(51) $$S_{1}=U_{0}\frac{\omega_{g}-\left(\omega_{g}\cos\omega_{g}t_{0}+j\omega \sin\omega_{g}t_{0}\right)\exp\left(-j\omega t_{0}\right)}{\omega_{g}^{2}-\omega^{2}}$$

**Figure 13:**
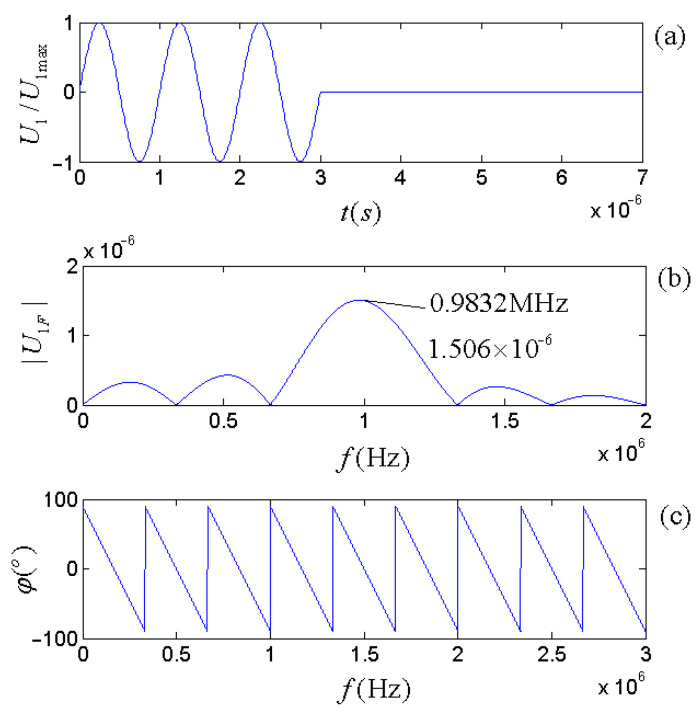
The gate-selection sinusoidal driving-voltage signal with three periods: (**a**) the waveform; (**b**) the amplitude spectrum; (**c**) the phase spectrum.

For the driving-voltage signal at a set of given parameters ($\omega_{g}=\omega_{1d}$, $U_{0}=$ 1 V, and $t_{0}=6\pi /\omega_{g}=6\pi /\omega_{1d}$), Figure 13 shows the time-domain waveform, amplitude spectrum, and phase spectrum. The frequency corresponding to the maximum amplitude spectrum (0.9832 MHz) is slightly lower than that of the gate-selection sinusoidal signal (1.000575 MHz).

When writing the gate-selection sinusoidal signal into a series of sinusoidal components, each sinusoidal component is taken as an independent excitation source corresponding to the parallel equivalent circuit in Part I of Figure 12.

**Figure 14:**
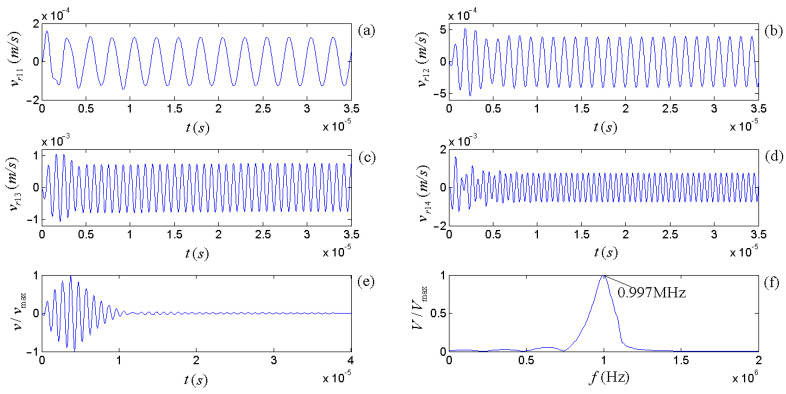
Figures (**a**–**d**) display the convolutions for several frequency components (0.4 $f_{1d}$, 0.8 $f_{1d}$, 1.2 $f_{1d}$, and 1.6 $f_{1d}$) and the corresponding electric–acoustic impulse response. Plot (**e**) shows the normalized accumulative output from the circuit network. And (**f**) is the normalized amplitude spectrum of the accumulative signal output.

All frequency components that excite the source transducer would make it radiate acoustic signals outward, for which their accumulation forms the acoustic wavelet emitted from the source transducer, as shown in Figure 14e. The calculated results show that when each frequency component in the driving-voltage signal excites the source, the radiated acoustic signal quickly transitions from the zero states to the steady sinusoidal vibration. The corresponding amplitude spectrum shown in Figure 14f shows that the center frequency of the radiated acoustic signal wavelet is 0.997 MHz, which is slightly lower than the center frequency of the gate sinusoidal driving-voltage signal.

**Figure 15:**
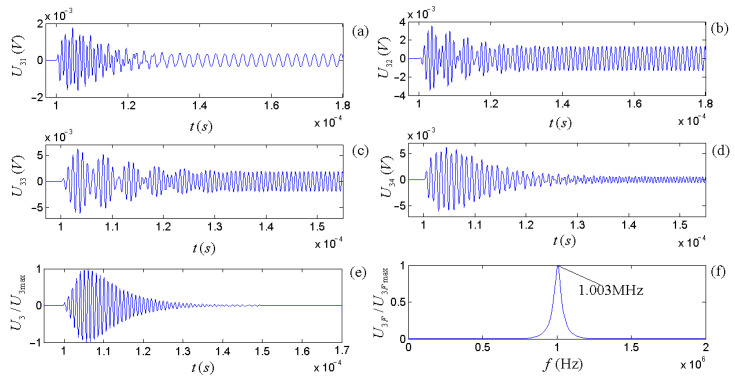
Plots (**a**–**d**) are the electric signals when the source is excited by several frequency components (0.4 $f_{1d}$, 0.8 $f_{1d}$, 1.2 $f_{1d}$, and 1.6 $f_{1d}$) of the gate sinusoidal driving-voltage signal. Plot (**e**) is the normalized time-domain waveform of the accumulative output. Plot (**f**) is the normalized amplitude spectrum of the accumulative signal output.

With respect to the integrated response characteristics of the source–receiver, Figure 15 shows the electric signal output at the receiver’s electric terminals when the source is excited by a gate-selection sinusoidal driving-voltage signal.

The calculated results show that compared with the acoustic signals of the radiated acoustic signal when the source is excited by each frequency component in the gate sinusoidal drive-voltage signal, the duration of the transient transition process of the corresponding output electric signal at the electric terminals of the receiver (see each circuit of Part III of the parallel network shown in Figure 12) becomes more prolonged, and the time domain waveform becomes more disorderly, as shown in Figure 14a–d.

The cumulative output of all parallel circuits in Part III in Figure 12, namely the electric signal outputted by the electric terminals at the receiver, is shown in Figure 15e. Compared with the acoustic signal wavelet radiated by the source, the oscillation duration of the electric signal at the receiver’s electric terminals becomes more extended, and the bandwidth of the corresponding amplitude spectrum becomes narrower. In addition, the amplitude of the frequency component far away from the center frequency of the receiver decreases significantly or disappears. The center frequency of the electric signal at the receiver’s electric terminals is 1.003 MHz, slightly higher than the acoustic signal radiated by the source due to the receiver’s acoustic–electric filtering effect on the received acoustic signal.

## 4. Experimental Measurement

**Figure 16:**
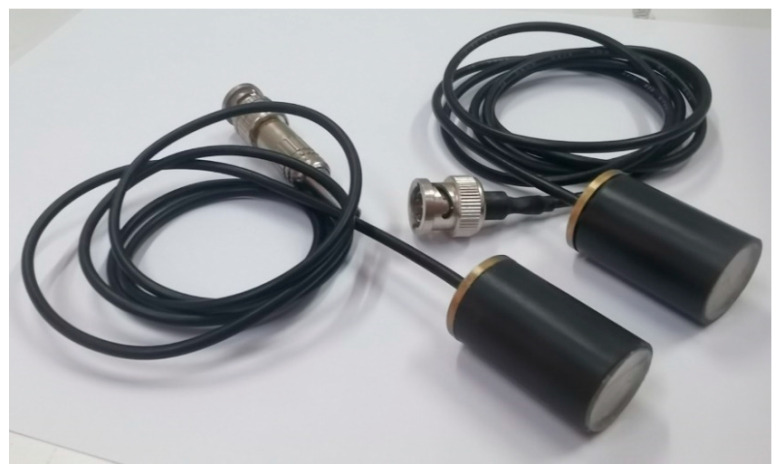
The physical image of the transducer.

To confirm the quality of our theoretical prediction, we selected two thin-wafer piezoelectric transducers with a thickness of 2 mm and a radius of 8 mm for experimental measurements in this paper. One acts as the source transducer, and another one works as the receiver. Table 1 illustrates that the material constituting the transducer is the manufactured PZT-5H and provides the corresponding physical parameters. Figure 16 shows the physical image of the transducer.

**Figure 17:**
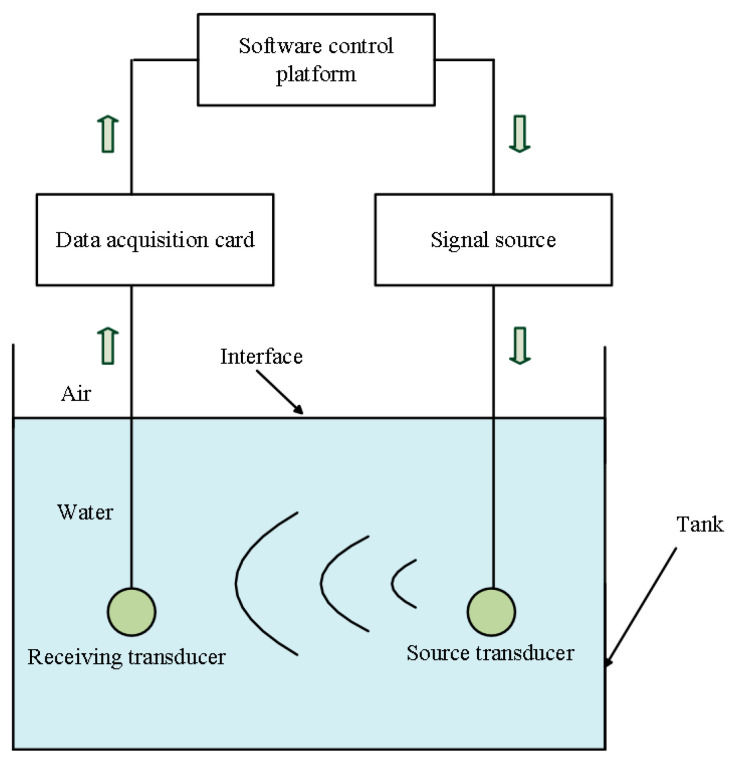
The structural chart of the experimental testing apparatus.

Figure 17 is the structural chart of hardware and software modules used in experimental testing. The hardware is composed of three modules: the mechanical module, the electronic hardware module, and the system’s software module. The mechanical module includes a stepper motor, a moving slide rail, a tank, and a steering gear. The electronic hardware module consists of a signal generator (AFG-125P, made by Gwinstek in China) with up to a bandwidth of 25 MHz and a resolution of 1 μHz, a microcontroller (STC12C5A60S2, made by STC MCU Limited in China) used to control the spatial position of the source and receiver, and a data collector (PXI-5922 made by NI in USA) with a sampling rate of 5–15 MHz and 16–24 bits. 

The system software based on the LabVIEW platform (Version of 2011) is used for measurement control, which consists of an electric-signal module, a display/storage module, and a slippage/rotation module.

We placed the source and receiver in a tank filled with water. The virtual panel on a computer screen gave instructions to place the two transducer electrodes opposite of one another at 15 cm apart. The control panel selects the gate-selection sinusoidal driving-voltage signal with an amplitude of 1 V, a gate width of 3 cycles, and frequencies of 0.3, 1.0, and 1.7 $f_{1d}$ to excite the source transducer. The measurement system collects and stores the signals from the receiver’s electric terminals. Finally, the calculated and measured data are compared on the MATLAB platform to verify the correctness of the model established in this paper.

**Figure 18:**
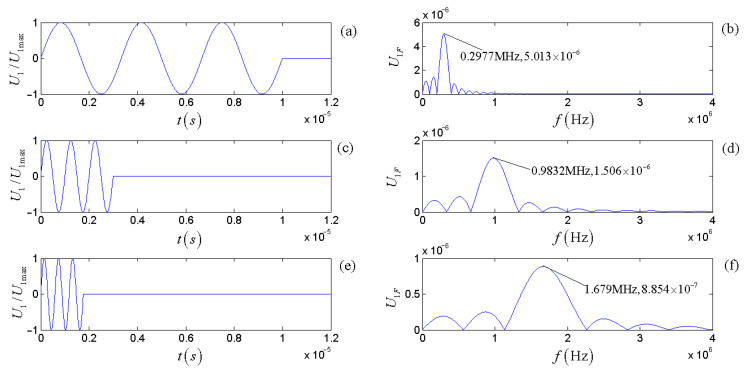
Time domain waveforms and amplitude spectra of three gate-selected sinusoidal voltage signals with different frequencies: (**a**,**c**,**e**) are the normalized time domain waveform; (**b**,**d**,**f**) are the corresponding amplitude spectra.

**Figure 19:**
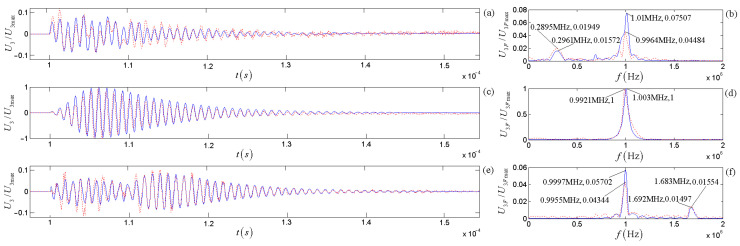
The electric signals at the receiver’s electric terminals under the excitations of the above three different gated sinusoidal electric signals: (**a**,**c**,**e**) waveforms; (**b**,**d**,**f**) amplitude spectra. Blue solid lines come from the theoretical calculation, and red dotted lines come from the experimental measurement.

Figure 18 shows the time-domain waves and amplitude spectra for the three selected driving-voltage signals. Figure 19 shows the calculated and measured time-domain waveforms and the corresponding spectra at the receiver’s electric terminals. Figure 19a,c,e show the normalized time-domain curve, and Figure 19b,d,f show the normalized frequency domain curve.

By comparing the red dotted line and solid blue line in Figure 18, the theoretically calculated results in this paper agree well with the experimental measurements. When the driving-voltage signal frequency’s center frequency is equal to or close to the transducer’s load center frequency, the signal amplitude at the receiver’s electric terminals reaches the maximum. When the center frequency of the driving-voltage signal is substantially different from the load center frequency of the source, the signal amplitude at the receiver’s electric terminals becomes smaller, and two peaks appear in the corresponding amplitude spectrum curves. One peak (maximum) is near the driving-voltage signal frequency’s center frequency, and the other is near the transducer’s load center frequency. 

Figure 19 also shows some minor differences between the theoretical calculations and experimental measurements, especially for the difference between sub-main peaks in the amplitude spectrum’s curves. 

The measured signal at the receiver’s electric terminals is from the contributions of the driving-voltage signal, the electric–acoustic conversion of the source, the physical property of the medium (water), and the acoustic–electric conversion of the receiver. At the same time, the calculated signal at the receiver’s electric terminals is from the contributions of the driving-voltage signal, the source’s electric–acoustic conversion, and the receiver’s acoustic–electric conversion. The most prominent peak reflects the source, receiver, or medium’s comprehensive frequency response. Meanwhile, the sub-main peak reflects the frequency property of the driving voltage signal used to excite the source.

## 5. Discissions and Concluding Remarks

By introducing two components (namely, radiation resistance and radiation mass) into the equivalent electric–mechanical circuit developed by predecessors, this paper established a new frequency-dependent dynamic electric–mechanical network for thin-wafer piezoelectric transducers. Because the newly introduced mechanical components are frequency functions, we extended the electric–mechanical circuit model to a parallel circuit network to treat multiple frequency signal transmissions. 

For the first time, we derived analytical expressions of electric–acoustic/acoustic–electric impulse responses and their transmission functions under the sinusoidal electric/acoustic signals excitation with any given frequency for thin-wafer piezoelectric transducers polarized in the thickness direction. 

The driving-voltage signal used to excite the source (in electric–acoustic conversion) is a signal wavelet with many frequency components. The particles inside the source transducer have mass and inertia. The vibration of particles inside the receiver and the electric signal at its electric terminals would still go through a transient process, even for a sinusoidal acoustic signal arriving at the receiver. The measured acoustic signal, i.e., the electric signal at the receiver’s electric terminals, will also not be a single-frequency sinusoidal signal. 

By carrying out analytical derivations, calculations, analyses, and experiment measurements, we conclude with the following remarks:

(a) The effects of the transducer’s geometric parameters on various frequencies related to the transducer’s properties presented in Figure 5, Figure 6, Figure 7 and Figure 8 are significant. Load and free-load center frequencies and free-load oscillation frequencies decrease significantly with increased thicknesses for all given radius ranges and a minimum change is observed with respect to the radius after a range of initial increases (Figure 5). The free-load center frequency is slightly higher than the load center frequency. The free-load center and oscillation frequencies are constant for a thin-wafer transducer with fixed physical and geometrical parameters.

In contrast, the loading center frequency varies slightly with the transducer’s steady-state vibration frequency (Figure 7 and Figure 8). With respect to the variation tendency of the frequency, the loading oscillation frequency increases with the (electric or acoustic) sinusoidal signal frequency (f) exerted on the transducer. The sinusoidal signal frequency has a more significant effect on the load oscillation frequency in the low-frequency range. There is a minimal impact on the load oscillation frequency in the higher frequency range (Figure 6).

(b) Concerning the frequency bandwidth, the source’s frequency response bandwidth is greater than the receiver’s bandwidth. Due to the electric–acoustic filtering of the source signal, the frequency bandwidth of the radiated acoustic signal is narrower than the driving-voltage signal. Meanwhile, due to the acoustic–electric filtering of the receiver, the frequency bandwidth of the electric signal at the receiver’s electric terminals, i.e., the measured acoustic signal, is narrower than that of the acoustic signal arriving at the receiver.
(c) Extending the single-frequency circuit network (Figure 3) to parallel multiple-circuit networks (Parts I and III of Figure 12) is necessary for the transitions of the multifrequency signal wavelet. Since thin-wafer transducers’ radiation resistance and mass are frequency functions, we may express the electric–acoustic (acoustic–electric) conversion excited by a multifrequency signal wavelet using a parallel transmission network, i.e., an equivalent mechanical–electric circuit network. The cumulative output from the parallel-circuit network represents the acoustic signal wavelet that is radiated outward by the source (and the measured acoustic signal wavelet, i.e., the electric signal wavelet at the receiver’s terminals).
(d) Comparing the integrated frequency response of the source–receiver with that of the source–medium–receiver, we can use the measured acoustic signal wavelet to obtain the medium’s physical properties and internal structure accurately.
(e) The center frequency shift of the transducer varies with the property mechanical loading (the medium around the transducer) and that of the driving-voltage signal to excite the transducer. If using free loading as a reference, the transducer exhibits higher sensitivity corresponding to lower frequency radiation resistance and radiation mass.
(f) Compared with Fa and Zhao’s previous works on thin spherical shells and cylindrical piezoelectric transducers [7,8], the time domain waveform obtained by a thin-wafer transducer vibrates for a longer period, and the corresponding amplitude spectrum has a narrower bandwidth, which is likely from the transducer’s different mechanical sizes, shapes, and polarization modes.

The new advancement discussed in this paper on the electric–acoustic conversion of the piezoelectric transducer circuit network should be worth some additional points considering the relevant published work in the literature. 

Piquette’s research has shown that there is a transient transition process in the electric–acoustic conversion of the piezoelectric transducer under the excitation of a sinusoidal electrical signal. The equivalent circuit given by Piquette missed two mechanical components, i.e., radiation resistance and radiation mass, for which their values are frequency functions. Without radiation resistance and radiation mass, the capacitive and inductive reactance generated by the capacitive and inductive components in the traditional equivalent circuit are frequency functions. Still, the values of the capacitive and inductive elements are independent of frequency. Therefore, that work missed carrying out further analyses on the transient transition process of the electric–acoustic conversion of multifrequency electrical signal (wavelet) excitation transducers [52,53]. Our paper introduced the radiation resistance and radiation mass into the equivalent circuit. These components are frequency functions, and the related and generated radiation force resistance and mechanical reactance are also frequency functions. 

Although Fa et al. introduced radiation resistance and radiation mass into the equivalent circuit of radially polarized thin spherical shell piezoelectric transducers [61,65] when multifrequency electrical signals stimulated the transducers, however, that work adjusted the transducer’s electric–acoustic conversion impulse response artificially and the values of radiation resistance and radiation mass constantly. Therefore, the obtained results from calculations and analyses have human subjective factors [65]. In the current paper, based on linear superpositions and the Fourier transform, the amplitude and phase spectra of the multifrequency electric-driving signal are discretized into many frequency components with different amplitude and initial phases. The equivalent circuits of a thin-wafer transducer polarized in the thickness direction form a parallel electric-acoustic conversion network corresponding to each electric driving-signal frequency (with different radiation resistances and radiation masses). Yet, the transmission network model proposed in this paper is the continuation and enhancement of the model of acoustic source transducers excited by multifrequency electrical signals reported by Fa et al. [65].

The method described in this paper differs from those of Mason and KLM for thin-disc piezoelectric transducers with respect to thickness polarization [49], and the transitional transient process generated by the electric–acoustic/acoustic–electric conversion of the transducer proposed in this paper is conceptually different from the transient characteristics of the transducer reported by Redwood [56]. In this paper, the spectrum corresponding to the abovementioned process is the intrinsic noise generated by the particles inside the transducer during the electric–acoustic/acoustic–electric conversion. We noted that the equivalent circuits of Mason and KLM used conceptual physical knowledge to describe the characteristics of thickness polarization piezoelectric transducers. In contrast, our new piezoelectric transducer model combines physics and signal transmission/processing knowledge to describe the characteristics of thin-wafer piezoelectric transducers.

Meanwhile, in the equivalent circuit models of Mason and KLM, some physical parameters of piezoelectric materials are assumed to be numerically complex, as shown in Tables 2 and 3 of reference [49], which is against physical nature. Even the propagation speed of the acoustic wave included an imaginary part, e.g., *v =* 2700(1 *+* 0.005*i*). In our new model, we applied all factual physical parameters (real numbers) of piezoelectric materials for calculations and analyses, which is physically more meaningful.

Considering the work of Sherrit et al. [49] on Mason and KLM Equivalent Circuits, the values of specific electrical components and mechanical components are independent of frequency, even though the impedance of these components can be frequency dependent. Similarly to ordinary equivalent circuits, the impedance values corresponding to the capacitive/inductive elements in the equivalent circuit are indeed frequency-dependent. In our new model, the two newly introduced mechanical components, i.e., radiation resistance and radiation mass, are numerically frequency functions [61,65]; the mechanical values of other electrical components are fixed and unchanged, which leads to obtaining a circuit of a different nature than the equivalent circuits reported in the previous literature; this is the basis for the establishment of a parallel electric–acoustic conversion transmission network, and it reflects the realistic situation of transducers better.

Additionally, the equivalent circuit of Mason and KLM is a six-terminal mechanic-electric network, i.e., four mechanical and two electrical terminals corresponding to two acoustic ports and one electrical port. Two acoustic ports correspond to the front and back surfaces of the thickness polarization transducer, and one electrical port corresponds to the two electrodes on the transducer’s front and back disk surfaces [49]. The disk’s back surface was tightly and entirely cemented with a thick rigid solid medium (assumed approximated that the back surface does not vibrate). Its front surface comprised a radiating surface coated with a thin insulating layer, considering that the thickness of the insulating layer was thin relative to the wavelength of the radiated acoustic wave, and its influence on the acoustic wave emitted by the transducer is ignorable. In our new physical model, we use the compression/tension deformation of a uniform rod (sheet) to describe the rear surface fixation and the front surface radiation of the thickness polarization thin-wafer transducer. By carrying this out, the above six-terminal machine–electrical equivalent circuit of Mason and KLM was simplified into a four-terminal equivalent circuit similar to but different from Camp’s thin ring piezoelectric transducer, noting that Camp’s equivalent circuit does not contain two frequency-related mechanical components, namely radiation resistance and radiation mass [63].

The transient transition process of particle vibrations inside the transducer reported in this paper also conceptually differs from the transient characteristics reported by Redwood [56]. Redwood’s qualitative description of the transient properties of piezoelectric transducers is as follows: “In the generation of ultrasonic waves a transient electrical signal is applied to the transducer and this produces a transient mechanical vibration, while in detection, the application of a transient mechanical signal produces an electrical vibration” [56]. If so, the transient electrical signal (applied to the transducer) and the transient mechanical vibration are essentially the electric driving signals of the acoustic source transducer and the acoustic signal radiated from the transducer. Then, both transient processes are signal wavelets within waveforms in the time domain with a specific time duration. In our model, when a transient mechanical signal is applied to the transducer, it generates a dynamic vibration. The concept of intrinsic noise generated from vibration particles inside viscous solids is introduced into the electric–acoustic and acoustic–electric conversion processes of thick-polarized thin-wafer piezoelectric transducers, which provides a theoretical basis for both extracting the intrinsic noise generated by the measured medium from the acoustic signal obtained in acoustic measurements and using the intrinsic noise to invert the physical characteristics and internal microstructure of the measured medium. It is essentially a vibrational wave propagating to the observation point to make its electrical terminals output a time-domain waveform with a specific duration with respect to the electric signal wavelet. 

We conclude that the updated parallel transmission network reported in this paper with the two new physical factors is more realistic relative to the physical nature of a multifrequency signal wavelet excitation transducer. It is applicable not only to conventional-sized but also to small-sized piezoelectric transducers, and it is universal in the descriptions of transducers.

## Figures and Tables

**Table 1:** Physical parameters of piezoelectric material PZT-5H.

**Parameter**	$C_{33}^{D}\left(N/m^{2}\right)$	$h_{33}\left(V/m\right)$	$\varepsilon_{33}^{S}/\varepsilon_{0}$	$\rho \left(\mathrm{kg}/m^{3}\right)$
**value**	$1.57\times 10^{11}$	$1.8\times 10^{9}$	1470	$7.5\times 10^{3}$

Symbol ($\varepsilon_{0}$) is the dielectric constant in a vacuum.
